# Prediction of Protein-Protein Interaction Sites by Multifeature Fusion and RF with mRMR and IFS

**DOI:** 10.1155/2022/5892627

**Published:** 2022-10-04

**Authors:** JunYan Zhang, Yinghua Lyu, Zhiqiang Ma

**Affiliations:** ^1^School of Information Science and Technology, Northeast Normal University, Changchun, 130024 Jilin, China; ^2^Graduate School, Northeast Normal University, Changchun 130024, Jilin, China

## Abstract

Prediction of protein-protein interaction (PPI) sites is one of the most perplexing problems in drug discovery and computational biology. Although significant progress has been made by combining different machine learning techniques with a variety of distinct characteristics, the problem still remains unresolved. In this study, a technique for PPI sites is presented using a random forest (RF) algorithm followed by the minimum redundancy maximal relevance (mRMR) approach, and the method of incremental feature selection (IFS). Physicochemical properties of proteins and the features of the residual disorder, sequence conservation, secondary structure, and solvent accessibility are incorporated. Five 3D structural characteristics are also used to predict PPI sites. Analysis of features shows that 3D structural features such as relative solvent-accessible surface area (RASA) and surface curvature (SC) help in the prediction of PPI sites. Results show that the performance of the proposed predictor is superior to several other state-of-the-art predictors, whose average prediction accuracy is 81.44%, sensitivity is 82.17%, and specificity is 80.71%, respectively. The proposed predictor is expected to become a helpful tool for finding PPI sites, and the feature analysis presented in this study will give useful insights into protein interaction mechanisms.

## 1. Introduction

Proteins interact with other proteins, DNA, RNA, and chemicals to play key roles in practically all biological events. Without initially defining the characteristics of contact sites, it is impossible to define the protein molecular structures. Proteins seldom act independently; instead, they are frequently part of a larger molecular network, with parts coordinated by complex protein-protein interaction (PPI) regulatory networks [1]. PPIs are important for practically every aspect of cellular function, including metabolic control, gene translation, DNA structure, and protein synthesis. Discovering the binding sites among interacting proteins, in particular, provides crucial information on the function of a protein and the elemental composition of associated proteins, assisting in the identification of biological targets and leading drug design. As a result, molecular recognition relies eavily on solving the issue of identifying interaction points [2].

Previously, a large number of properties that have some predictive potential for interfaces have already been identified [3, 4]. They are composed of three methods: the first uses sequence information alone to predict protein interfaces, the second applies structural information to improve sequences that are then used to build predictors, and the third method predicts using only 3D structure and sequence information [5].

Several approaches for predicting PPI sites have already been presented. Based on the utilized protein characteristics, they can be divided into three types. The first class's methods are solely dependent on sequence information. Ofran and Rost [6] employed a dataset, which consisted of 1,134 chains in 333 complexes and 59,559 touching residues. In their study, they correctly identified the PPI site in 20% of the complexes with 70% of their predictions being correct. The second class of methods combines secondary structure and sequence information. Zhou and Shan [7] established a predictor that was trained on 615 nonhomologous complex-forming protein pairs and tested on 129 nonhomologous complex-forming protein pairs. In their study, 70% of interface residues were properly predicted. Wang et al. [8] obtained an accuracy of 65.4% and a correlation value of 0.297 using a nonredundant data set of 69 protein chains. The third class uses 3D information of structures sequence information to make predictions. Aytuna et al. [9] proposed a technique that was evaluated sequentially nonredundantly on 67 interfaces and a nonredundant dataset of 6,170 protein structures. Public databases such as the Biomolecular Interaction Network Database, the database of Interacting Proteins, and PDB validated the majority of the 62,616 probable relationships. Sikic and Tomic [10] suggested a sequence-based prediction approach that has an 84% accuracy rate and a 26% recall rate. When structural information is included, prediction performance improves to 76% precision and 38% recall.

Several machine learning techniques have been developed to identify PPI sites based on various types of information. The author in [11] evaluated using a support vector machine (SVM) 50 randomly selected proteins and reported 60.6%, 53.4%, and 0.243 sensitivity, specificity, and MCC, respectively. Bradford et al. [12] used a Bayesian network to predict PPI sites with an 82% accuracy on a dataset of 180 proteins. Although great achievements have been made, the results still face difficulties to address the problem of predicting interaction sites [13]. Challenges remained to be overcome. First, the key biological features for properly defining protein-protein interaction sites have yet to be thoroughly identified. There is no way to identify interaction interfaces from other surface patches using a single parameter. As a result, several studies were conducted for the prediction of PPI sites using a combination of features. [14]. Second, present approaches for predicting PPI residues frequently rely on information taken directly from amino acid sequences, which is insufficient to extract all relevant information. Finally, in the prediction of protein interaction sites, a skewed class distribution problem is common [15]. A protein's number of interaction sites is generally substantially lower than its number of noninteracting sites. Overfitting and poor performance are common outcomes of such an imbalance, which is especially true for data in the interacting class [16].

In this study, a new approach is presented for identifying PPI sites, combining RF and mRMR, followed by IFS. To predict PPI sites, we used physiochemical properties, sequence conservation, residual disorder, secondary structure, and eleven 3D structural features. The datasets used in this study are derived by following methods. Firstly, the individual proteins are extracted from a set of 70 protein-protein heterocomplexes. Proteins with sequence identity less than 30% are subsequently obtained after removing redundant proteins and molecules with less than 10 residues. Some proteins that are not available in HSSP and DSSP programs are also omitted. As a result, 99 polypeptide chains are extracted from 54 heterocomplexes, which can be grouped into six categories. The categories and the number of representatives in each category (the values in the parentheses) are as follows: antibody antigen (29), protease inhibitor (19), enzyme complexes (14), large protease complexes (8), G proteins (13), and miscellaneous (16). The DSSP program works by calculating the most likely secondary structure assignment given the 3D structure of a protein. It does this by reading the position of the atoms in a protein followed by calculation of the H-bond energy between all atoms. The algorithm will discard any hydrogens present in the input structure and calculates the optimal hydrogen positions by placing them at 1.000 Å from the backbone N in the opposite direction from the backbone C=O bond. The best two H-bonds for each atom are then used to determine the most likely class of secondary structure for each residue in the protein. The surface residues are defined based on their relative solvent accessible surface area (RASA), which is calculated by the DSSP program. A residue is considered as a surface residue if its RASA is greater than 25%. A total of 13,771 surface residues are collected from all these polypeptide chains. Furthermore, a surface residue is defined to be an interface residue if its calculated ASA in the complex (CASA) is less than that in the monomer (MASA) by at least 1 Å^2^. This way, the number of protein-protein interaction sites is about 10% (2,828 residues) of the whole set of residues contained in the selected polypeptide chains (27,442 residues). Therefore, a total of 2,828 interaction sites are obtained as positive samples and 24614 noninterface residues are defined as negative samples. We predicted the PPI sites by both sliding window and patch analysis methods using the datasets; the results showed that the accuracy of the sliding window is superior to patch analysis. The proposed predictor outperformed numerous other state-of-the-art predictors in terms of accuracy, sensitivity, and specificity, with an average prediction accuracy of 81.44%, a sensitivity of 82.17%, and specificity of 80.71%.

The rest of the manuscript is ordered as: Section 2 provides a detailed description of the proposed method and data collection. Different feature selection methods and classification algorithms are discussed in this section.  Section 3 illustrates the results, and the conclusion is presented in Section 4. All authors contributed equally in this research.

## 2. Methodology

**Figure 1:**
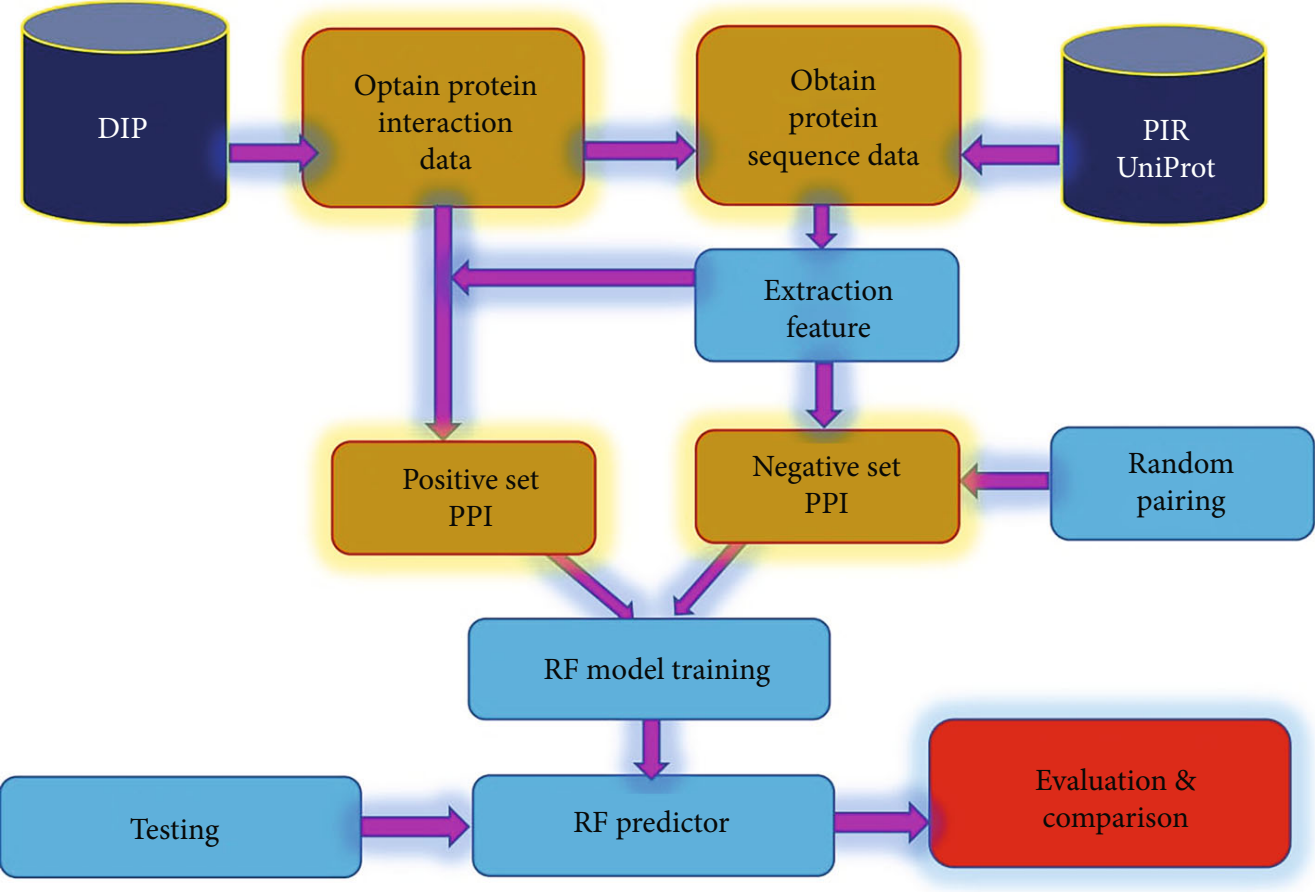
Propose methodology.

The proposed methodology is given in Figure 1.

### 2.1. Dataset

In this study, the PPI datasets were retrieved from the dataset developed by Jones and Thornton [17]. A total of 99 polypeptide chains were recovered from the 54 heterocomplexes in the sample. All proteins were taken from the Protein Data Bank (PDB) at http://www.pdb.org.

First, we determined the RASA of all surface residues detected by the DSSP algorithm [18]. If a residue's RASA is greater than 25%, it is classified as surface residue. Amino acid solvent exposure is essential for investigating and forecasting protein interaction and function. Half-sphere exposure (HSE), contact number (CN), residue depth (RD), accessible surface area (ASA), and relative accessible surface area are only a few of the different elements that make up solvent exposure features (RASA). Predicting protein-protein interaction hotspots has been done widely and successfully using the available solvent [19]. Solvent accessibility has the drawback of being unable to reveal any information regarding completely submerged leftovers. Half-sphere exposure (HSE), in contrast to conventional solvent access, can more accurately depict the local surroundings of the target residue from a different angle. The average atom depth of target residue atoms is represented by RD, and the number of residues within a given radius is represented by CN [20]. There were 13,771 surface residues. If the RASA in the complex (CASA) is less than the RASA in the monomer (MASA) by at least one unit, the surface residue is classified as an interface residue [21]. This way, the number of PPI sites in the selected polypeptide chains is about 10% (2,811 residues) of the total number of residues (27,442 residues). As a result, positive samples include 2,811 interaction sites, whereas negative samples have 24614 noninterface residues.

The unbalanced sample size will cause overfitting of the sample with a large proportion; that is, the prediction is biased toward a classification with a larger number of samples, which will reduce the applicability of the model. The processing method at the data level is sampling. Undersampling, oversampling, and combined methods are three common and widely used approaches. Eight different methods from an unbalanced-learning library can be adopted to deal with the unbalanced data. These eight methods include SMOTE, ADASYN, BorderlineSMOTE, SVMSMOTE, ClusterCentroids, NearMiss, SMOTEENN, and SMOTETomek. The data sets processed by the above methods were subjected to the same subsequent experimental operations, so as to compare the results obtained by different processing methods, and to select a method that is more suitable for processing antioxidant proteins.

### 2.2. Feature Extraction

A wide range of characteristics was used in our experiment to classify protein interaction sites, including sequence features, secondary structure features, and 3D structural properties which are described as follows:

#### 2.2.1. Sequence Feature

*(1) Amino Acid Factors*. Amino acids have several attributes, and there is a related database that has already organized and recorded the various attributes of amino acids, including physicochemical properties and biochemical properties [21]. AAIndex is a database that contains a quantitative index of numerous physicochemical and biological characteristics of amino acids. AAIndex was subjected to multivariable statistical analysis by Atchley et al. [22], which yielded five numeric attribute patterns: polarity, molecule volume, secondary structure, codon diversity, and electrostatic charge. These are the five numerical pattern scores used in this study. Each amino acid in a protein was encoded using these five amino acid parameters. Atchley factors are used to encode the physicochemical properties of amino acids. Each amino acid was represented by five Atchley factors, namely, polarity, codon diversity, secondary structure, molecular volume, and electrostatic charge. These five patterns or multidimensional indices were interpreted as follows: factor *I* = a complex index reflecting highly intercorrelated attributes for polarity, hydrophobicity, solvent accessibility, etc.; factor II = propensity to form various secondary structures, e.g., coil, turn, or bend versus alpha helix frequency; factor III = molecular size or volume, including bulkiness, residue volume, average volume of a buried residue, side chain volume, and molecular weight; factor IV = relative amino acid composition in various proteins, number of codon coding for an amino acid, and amino acid composition; factor V = electrostatic charge including isoelectric point and net charge. A set of factor scores arising from these analyses provide a multidimensional index positioning each amino acid in these major interpretable patterns of physiochemical variation.

*(2) PSSM Conservation Scores*. Posttranslational modifications are common in the conservation protein regions, and that is why evolutionary conservation is vital for biological function. The position-specific score matrix (PSSM) was employed to measure the preservation of each amino acid in a protein sequence. For each residue, a 20D vector was utilized to represent the probability of conservation against mutations to 20 distinct amino acids. The position-specific scoring matrix (PSSM) [23] is a matrix composed of all such twenty-dimensional vectors for a particular peptide. PSSM conservation scores are obtained from position-specific iterative BLAST (PSI-BLAST) with parameters *j* = 3 and *h* = 0.001. Moreover, the alignment database is Swisspro [24].

#### 2.2.2. Secondary Structure Feature

*(1) Secondary Structure*. The secondary structure of the relevant residues may impact the protein structures that play critical roles in protein function and the posttranslational modifications of certain residues [24]. In this study, we also employed solvent accessibility and secondary structure to encode each peptide in our investigation. The predictor SSpro4 [25] predicted solvent accessibility and secondary structure [26]. SSpro4 data was encoded using the letters ‘E' for strand, ‘H' for helix, and ‘C' for other. A 3D binary vector was used to convert these words into numeric vectors: 100, 010, and 001, respectively. Because buried residues are never present in a protein interface, we deleted all peptides in all samples that were centered on a predicted buried residue.

*(1) Disorder Score*. Interaction sites, which are critical loci for numerous protein-protein interactions like methylation and phosphorylation, are frequently abundant in intrinsic disorder areas. As a result, such areas are crucial for protein structure and function [27]. As a consequence, we encoded the peptides using the structural disorder of the residues in the sequence. VSL2, which can reliably predict both short and long disordered areas in proteins, was used to compute a disorder score for each residue in a given protein sequence [28].

#### 2.2.3. 3D Structural Features

The method of using the 3D structural feature plays an important role in promoting the effects of PPI site prediction [29]. We also employed this method in our study; based on the work of Sikic et al. [10], we selected 7 different features, namely, ASA, RASA, DPX, CX, AS, and SC, as well as another feature named Hydrophobicity. We used the program Protein structure and Interaction Analyzer (PSAIA) and the program Surface Racer [18] to fetch these 3D architectural features from the PDB database.

*(1) Features from PSAIA*. PSAIA is a program that calculates geometric parameters for a large number of protein structures to anticipate and explore protein-protein interaction sites [30]. It is possible to determine the following geometry parameters: The Kyte and Doolittle scale assigns a hydrophobicity value to each residue [31, 32].

*(2) Features from Surface Racer*. Research has shown that the way components of the large molecular surface interact with solvent and tiny solutes in solution determines protein stability and solubility [33]. As a result, one of the most critical factors in determining the structure of large molecules and their function is the macromolecular surface. In this study, we also took into account Molecular Surface Area and Surface Curvature. Program Surface Racer has estimated these characteristics based on the PDB database. These features were predicted by Program Surface Racer from the PDB database.

### 2.3. The Feature Space

We used 40 features for each residue in a protein segment, including 5 amino acid factor features, 20 PSSM conservation score features, 3 secondary structure features, 1 disorder feature, and 11 3D structural features from the PDB data.

In this study, we use three different kinds of ways to build feature space. Firstly, we use a single residue. Secondly, using window sliding, we were able to obtain N-residue protein segments centered on the indicated PPI residue, with *n* residues upstream and *n* residues downstream of the interaction site. For the peptides with lengths less than *N* amino acid residues, we complemented it with “X”, where 1 ≤ *n* ≤ 19, and *N* = 2*n* + 1. Thirdly, we extracted a patch-based model to characterize every residue. Each patch was made up of a center residue and its *m* − 1 nearest spatial residues, where 10 ≤ *m* ≤ 25. The nonsurface residues are ignored in the present study, which solely deals with surface residues.

### 2.4. mRMR Method

To value the importance of each feature, we employed the mRMR technique [34]. The mRMR technique grades features based on their association to the target as well as feature redundancy. Mutual information (MI), which estimates the degree to which one vector is connected to another, was used to quantify both relevance and redundancy. The MI can be expressed using

(1) $$\begin{array}{c} \text{I}\left(x,y\right)=\underset{i,j}{\sum }p\left(x_{i},y_{j}\right)\log\frac{p\left(x_{i},y_{j}\right)}{p\left(x_{i}\right)p\left(y_{j}\right)}, \end{array}$$

where *x* and *y* represent the two random vectors,  *p*(*x*) and *p*(*y*) are the marginal probabilistic densities, and  *p*(*x*_*i*_, *y*_*j*_) is the joint probabilistic density. Mutual information can well describe the selected features and the relationship between the output categories. If the output category has selected features and mutual information, the more evidence that the characteristics comprise information categorization, the more effective it will be for classification and recognition [7]. We can select contribution to feature subset categorization by computing mutual information between the characteristics and the type and characteristics. The bigger the redundancy, the better the characteristic. The smaller the correlation, the better the feature will be represented. We determined that we could extract data from the results using the MIQ approach. We assume that the set *K*_*m*_ is the existing selected feature set composed of *m* features, and the set *K*_*n*_ is the feature set to be selected having *n* features.

(2) $$\begin{array}{c} D=I\left(f,c\right), \end{array}$$

where *D* is the association between the feature *f* in *K*_*m*_ and the class *c*, and *R* is the association between the feature *f* in *S*_*m*_ and all features in *S*_*n*_, and *R* can be calculated by

(3) $$\begin{array}{c} R=\frac{1}{m}\underset{fi\in K_{m}}{\sum }I\left(f,f_{i}\right). \end{array}$$

So the feature *f*_*i*_ in the set *K*_*n*_ can be calculated as

(4) $$\begin{array}{c} \underset{f_{j}\in K_{n}}{\max}\left[I\left(f_{j},c\right)-\frac{1}{m}\underset{f_{i}\in M}{\sum }I\left(f_{j},f_{i}\right)\right],\;\left(j=1,2,\cdots ,n\right). \end{array}$$

In this work, we used the mRMR program which is publicly available at http://penglab.janelia.org/proj/mRMR/.

### 2.5. Random Forest

In this study, RF was used as the prediction engine, and the default settings were used. An RF is an ensemble predictor composed of several decision trees. A new query sample coded by an input vector is placed into each of the forest's trees to classify it. A projected class is provided by each decision tree [35]. The class with the most votes will be chosen as the random forest's anticipated class.

In this work, we employed the RF program embedded in the Weka package for classification, and the Weka package is openly available at http://www.cs.waikato.ac.nz/ml/weka/index_downloading.html.

### 2.6. Incremental Feature Selection

Based on the data list, we selected the optimal characteristic set with IFS. It ranks each eigenvalue on the list by scores from high to low. Moreover, every single time we chose the first *i*(1 ≤ *i* ≤ 40) with feature concentration as feature subset. We derived consequences with the RF classifier; eventually, we obtained 40 feature subsets with 40 prediction results and selected the character subset with the highest value of MCC as the optimal feature subset.

(5) $$\begin{array}{c} F=\left\{f_{1}^{\prime },f_{2}^{\prime },f_{3}^{\prime },\cdots ,f_{i}^{\prime }\right\}. \end{array}$$

### 2.7. Prediction Engine and Assessment

We used 10 cross-validation and different evaluation metrics to assess each predictor's performance. The evaluation metrics included sensitivity, precision, specificity, accuracy, and MCC (Mathews correlation coefficient) were used. These measures were calculated as shown below:

(6) $$\begin{array}{c} \left\{\begin{array}{c} \text{sensitivity}=\frac{\text{TP}}{\text{TPN}}\text{where TPN}=\text{TP}+\text{FN}\\ \text{precision}=\frac{\text{TP}}{\text{TFP}}\text{where TFP}=\text{TP}+\text{FP}\\ \text{specificity}=\frac{\text{TN}}{\text{TN}+\text{FP}}\\ \text{accuracy}=\frac{\text{TP}+\text{TN}}{\text{TP}+\text{TN}+\text{FP}+\text{FN}}\\ \text{MCC}=\frac{\text{TP}*\text{TN}-\text{FP}*\text{FN}}{\sqrt{\left(\text{TP}+\text{FP}\right)\left(\text{TP}+\text{FN}\right)\left(\text{TN}+\text{FP}\right)\left(\text{TN}+\text{FN}\right)}}, \end{array}\right. \end{array}$$

where TP denotes true positive, TN denotes true negative, FP denotes false positive, and FN denotes false negative.

## 3. Results and Discussion

### 3.1. Performance Evaluation on Different Feature Space Methods

In this study, we used three kinds of the classical method of constructing feature space, namely, single residue, nonoverlapping sliding window, and overlapped patch-based method, on the spatial structure. We compared all three methods using three machine learning models. The prediction result of the single residue of feature space is shown in Table 1.

**Figure 2:**
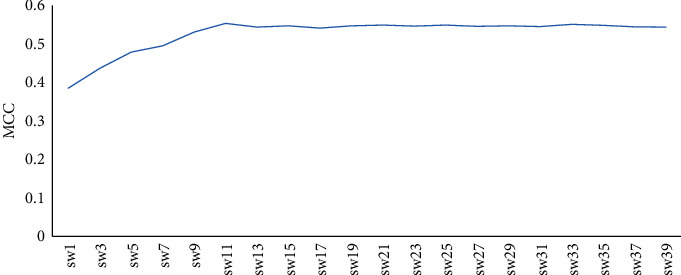
The prediction results with the feature space of the sliding window.

We can see that the predicting outcomes are with lower accuracy and sensitivity but higher specificity. It shows that the problem of the distinctiveness of feature from the feature space of the single residue has not been well addressed. The second way to build feature space is based on the sequence of sliding window, window length *N* = 2*n* + 1(1 ≤ *n* ≤ 19). The prediction results are shown in Figure 2.

It can be observed that the curve shows an upward trend and the overall curve is smooth. When the length of the sliding window increased, the prediction results are better. When the window length is stable at 11, the curve is smooth. When the length is 11, it reaches the maximum value based on the method of the sliding window. For a window of length 11, the results of the evaluation index *t* are as shown in Table 2. The sensitivity, precision, specificity, accuracy, and MCC obtained are 0.463, 0.834, 0.974, 0.870, and 0.553, respectively.

**Figure 3:**
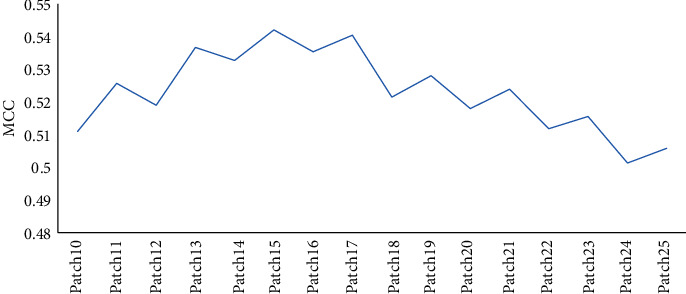
The prediction results with the feature space of the patch.

The third way to build feature space is using the patch-based method. We extracted a patch-based model to characterize every residue. Each patch was made up of a center residue and its *m* − 1 nearest spatial residues, 10 ≤ *m* ≤ 25. The prediction results are shown in Figure 3.

We can see from Figure 3 that the curve is overall steeper and presents a rising tendency, and when the number of residues in the patch increased, the prediction results are better. When we take the number of residue from the patch with 15, the MCC reaches its maximum value. Based on the method of the patch, we take the window length with 15 and take this outcome as the result of this method (as shown in Table 3). The sensitivity, precision, specificity, accuracy, and MCC obtained are 0.471, 0.793, 0.968, 0.866, and 0.542, respectively.

To provide a comprehensive analysis of the three methods, Table 4 shows the comparative results of the three methods in terms of sensitivity, specificity, accuracy, and MCC.

Based on the results of the 3 methods, it is evident that the predicted outcomes of sliding windows and patch methods are ideal than the single residue. Hence, this study employed the method of the patch and sliding windows to predict the results.

### 3.2. Performance Evaluation with Different Parameters

**Figure 4:**
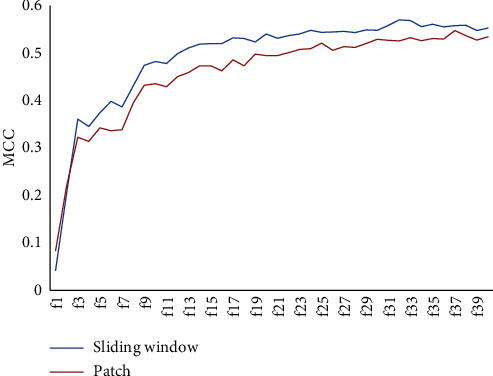
Sliding window and patch mRMR results.

Figure 4 shows the prediction results when the features are selected using mRMR. Out of all features, 40 features were selected using mRMR. Two methods of feature space were employed, namely, the sliding window and patch-based feature space method. For the sliding window, the length of the sliding window was set to 11, and for the patch-based method, 15 is used.

We have separately established 40 feature subsets for window size 11 and patch 15 with the method of IFS and then obtained the prediction results as shown in Figure 4. The increasing tendency is obvious when we take the number of features from 1 to 9, then the curve tends to smooth and the MCC reaches its maximum value when the number of the features is 32. Similarly, the increasing tendency is obvious when we choose the number of features from 1 to 11, then the curve tends to smooth and the MCC reaches its maximum value when the number of the features is 37. The value of sensitivity, precision, specificity, accuracy, and MCC reported are 0.449, 0.831, 0.977, 0.869, and 0.547, respectively. The comparative results of the sliding window with patch ways show that the sliding window has more exact results than a patch.

We can conclude from Table 5 that the value from the optimal feature subset with window size 11 is higher than the MCC and sensitivity of patch 15.

### 3.3. Performance with Different RF Parameters

The RF (retention factor) is defined as the migration distance of the protein through the gel divided by the migration distance of the dye front [36]. The distance should be measured from the top of the resolving gel to the band of interest. An RF value is the distance in millimeters the amino acid traveled over the distance that the solvent traveled in millimeters. The RF values in thin layer chromatography are affected by the absorbent, the solvent, the chromatography plate itself, the application technique, and the temperature of the solvent and plate [37].

**Figure 5:**
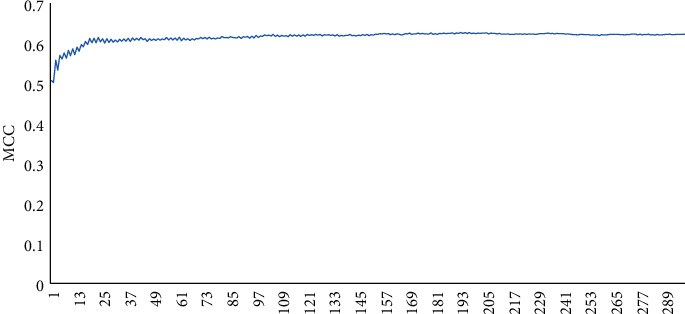
The influence of TREE number on the result.

The default parameters of the random forest were used along with 10-fold cross-validation. To achieve optimized results, we repeat the experiment many times for each parameter and select the optimal result.  Figure 5 shows the results for different tree numbers. It can be observed that with an increase in the number of RF trees, the MCC values increase from 1 to 27. After number 27, the line becomes straight.

To focus on both efficiency and accuracy, we selected the 197 fattest trees as samples and generated the results. The sensitivity, precision, specificity, accuracy, and MCC obtained are 0.553, 0.839, 0.973, 0.887, and 0.621, respectively, as shown in Table 6.

**Figure 6:**
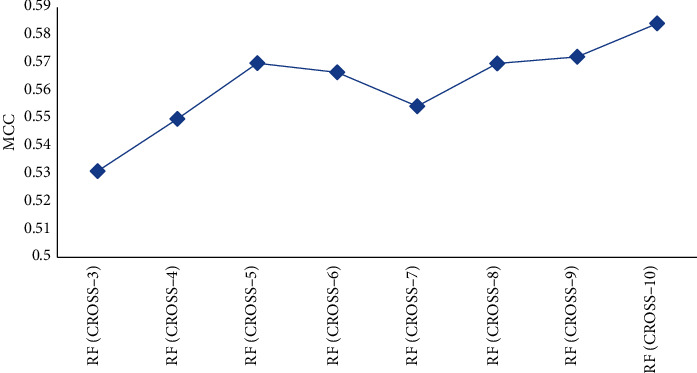
The influence of cross-validation adjustment on the result.

Figure 6 shows the results of cross-validation. We have selected different numbers of folds including 3 to 10. All the MCC values show an upward trend; therefore, we employed the 10-fold cross-valuation, and the sensitivity, specificity, precision, accuracy, and MCC obtained are 0.551, 0.976, 0.855, 0.877, and 0.636, respectively (Table 7).

In this study, a total of 2,811 interaction sites are obtained as positive samples and 24614 noninterface residues are defined as negative samples. Positive samples are far less than negative samples; it will affect the result of prediction. To eliminate the imbalance of the positive and negative samples, we selected the same number of negative as positive samples repeatedly and combined them as individual subdatasets, and then the average results are taken as the last prediction results.

**Figure 7:**
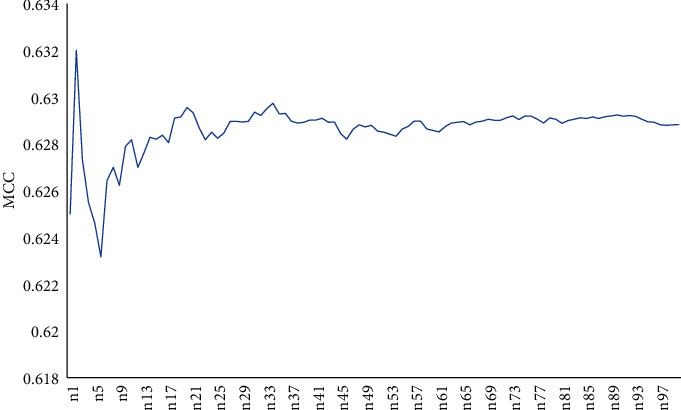
The results of 100 times the average.

Figure 7 shows that the MCC scores obtained from random sampling for a hundred times and the sensitivity, precision, specificity, accuracy, and MCC reported are 0.822, 0.81, 0.807, 0.814, and 0.667, respectively. To verify the effectiveness of disposing unbalanced sample data, we compared the prediction results of the raw data and processed unbalanced sample data, as shown in Table 8.

From the above table, we can see that compared with the raw data the sensitivity of the processed balanced sample data has increased significantly. Sensitivity plays an important in evaluating the prediction results. MCC is a general index, from the omnibus MCC index that shows balanced number data set can get the highest MCC number. Therefore, we can conclude that the result of this paper is effective.

### 3.4. Performance Comparison with Existing Predictors

In previous studies, some researchers predicted interaction sites only from a single feature and method of constructing the single feature space rather than the ways used in our experiments [38, 39]. To further evaluate the effectiveness of the prediction method in this work, three additional experiments are implemented to predict interaction sites by utilizing the methods of Wang et al.'s [8], Nguyen and Rajapakse's [27], Ofran and Rost's [6] studies and the present study. The results of the four experiments are reported in Table 9.

It clearly shows that the performance of the proposed method outperforms the other three methods especially in terms of sensitivity and the MCC values. Since higher sensitivity means a better prediction in positive classes, it is very useful for correcting the identification of interface residues. The MCC value represents the composite index; the higher the MCC value, the better the overall performance of the predictor. This validates that the approach presented in this study is competent to the PPI sites.

## 4. Conclusion

Proteins play a crucial role in cell life activities, particularly in terms of predicting protein interaction sites, which allows us to get a better understanding of protein function and molecular recognition. This study established a new approach for predicting PPI sites and employed three distinct types of feature collection methods including sequence signatures, secondary structure feature, and 3D structural features. In addition, the model is designed with three features at the same time to evaluate the sliding window and patch methods based on the same public datasets. Within the PPI area, the proposed technique examines not only the physical properties of each amino acid but also sequence conservation information and residue disorder status. The PDB dataset is used to analyze solvent accessibility, secondary structure, and 3D structural properties. It also demonstrates that the sliding window classification accuracy is greater than the patch-based feature space method. In the proposed method, 32 characteristics were employed and an MCC value of 62.88% was achieved. The experiments results show that the proposed method is effective in addressing the problem of predicting the protein interaction site.

## Figures and Tables

**Table 1:** For a single residue of feature space prediction results.

Dataset	Sensitivity	Specificity	Precision	Accuracy	MCC
Single	0.267	0.980	0.787	0.835	0.390

**Table 2:** For sliding windows of feature space prediction best results.

Dataset	Sensitivity	Specificity	Precision	Accuracy	MCC
SW11	0.463	0.974	0.824	0.870	0.553

**Table 3:** For a patch of feature space prediction best results.

Dataset	Sensitivity	Specificity	Precision	Accuracy	MCC
Patch15	0.471	0.968	0.793	0.866	0.542

**Table 4:** The ideal results compared from single residue, sliding window, and patch.

Dataset	Sensitivity	Specificity	Precision	Accuracy	MCC
Single	0.267	0.980	0.778	0.835	0.390
SW11	0.463	0.974	0.824	0.870	0.553
Patch15	0.471	0.968	0.793	0.866	0.542

**Table 5:** Sliding window and patch IFS result.

Dataset	Sensitivity	Specificity	Precision	Accuracy	MCC
SW11_32	0.480	0.975	0.833	0.874	0.570
Patch15_37	0.449	0.977	0.831	0.869	0.547

**Table 6:** The influence of the TREE number on the result.

Dataset	Sensitivity	Specificity	Precision	Accuracy	MCC
Default trees	0.498	0.975	0.838	0.878	0.584
197_trees	0.550	0.976	0.854	0.889	0.627

**Table 7:** The influence of cross-validation adjustment on the result.

Dataset	Sensitivity	Specificity	Precision	Accuracy	MCC
10-fold cross-validation	0.551	0.976	0.855	0.877	0.636

**Table 8:** The results over imbalanced and trimmed data.

Dataset	Sensitivity	Specificity	Precision	Accuracy	MCC
Imbalanced	0.551	0.976	0.855	0.877	0.636
Trimmed	0.822	0.807	0.810	0.814	0.667

**Table 9:** Performance comparison with other methods.

Methods	Sensitivity	Specificity	Accuracy	MCC
Wang et al. [8]	0.698	0.666	0.729	0.230
Nguyen and Rajapakse [27]	0.436	0.926	0.803	0.349
Ofran and Rost [6]	0.763	0.786	0.863	0.376
Proposed method	0.822	0.807	0.814	0.667

## Data Availability

The data used to support the findings of this study are included within the article.
